# Retinopathy of Prematurity in the 21st Century and the Complex Impact of Supplemental Oxygen

**DOI:** 10.3390/jcm12031228

**Published:** 2023-02-03

**Authors:** Sarah H. Rodriguez, Anna L. Ells, Michael P. Blair, Parag K. Shah, C. Armitage Harper, Maria Ana Martinez-Castellanos, S. Grace Prakalapakorn, Erima Denis, Rebecca C. Lusobya, Mark J. Greenwald, Sherwin J. Isenberg, Scott R. Lambert, Yvonne E. Vaucher, Ann Carroll, Lucy Namakula

**Affiliations:** 1 Department of Ophthalmology and Visual Science, University of Chicago Medical Center, Chicago, IL 60637, USA; 2 Calgary Retina Consultants, Department of Surgery, University of Calgary, Calgary, AB T2H 0C8, Canada; 3 Retina Consultants, Ltd., Des Plaines, IL 60016, USA; 4 Department of Vitreous & Retina, Aravind Eye Hospital, Coimbatore 641014, India; 5 Austin Retina Associates, Austin, TX 78705, USA; 6 Asociación Para Evitar la Ceguera En Mexico, Hospital “Luis Sáchez Bulnes” I.A.P. Department of Pediatric Retina, Mexico City 04030, Mexico; 7 Department of Ophthalmology and Pediatrics, Duke University Medical Center, Durham, NC 27710, USA; 8 Duke Global Health Institute, Duke University Medical Center, Durham, NC 27710, USA; 9 Lubaga Hospital, Kampala P.O. Box 14130, Uganda; 10 Masaka Regional Referral Hospital, Kampala P.O. Box 7272, Uganda; 11 Department of Ophthalmology, School of Medicine, College of Health Sciences, Makerere University, Kampala P.O. Box 7072, Uganda; 12 Mulago National Referral Hospital, Kampala P.O. Box 7051, Uganda; 13 Jules Stein Eye Institute, David Geffen School of Medicine, University of California, Los Angeles, CA 90095, USA; 14 Department of Ophthalmology, Stanford University School of Medicine, Palo Alto, CA 94305, USA; 15 Division of Neonatology, Department of Pediatrics, University of California San Diego, San Diego, CA 92037-7774, USA; 16 Independent Researcher, Roseville, MN 55113, USA; 17 St. Francis Hospital Nsambya, Kampala P.O. Box 7146, Uganda

**Keywords:** retinopathy of prematurity, oxygen-associated retinopathy of prematurity, oxygen, global health, ophthalmology, global ophthalmology, childhood blindness, pediatric blindness, retrolental fibroplasia, oxygen-induced retinopathy

## Abstract

Retinopathy of prematurity (ROP) is a leading cause of childhood blindness. Not only do the epidemiologic determinants and distributions of patients with ROP vary worldwide, but clinical differences have also been described. The Third Edition of the International Classification of ROP (ICROP3) acknowledges that aggressive ROP (AROP) can occur in larger preterm infants and involve areas of the more anterior retina, particularly in low-resource settings with unmonitored oxygen supplementation. As sub-specialty training programs are underway to address an epidemic of ROP in sub-Saharan Africa, recognizing characteristic retinal pathology in preterm infants exposed to unmonitored supplemental oxygen is important to proper diagnosis and treatment. This paper describes specific features associated with various ROP presentations: oxygen-induced retinopathy in animal models, traditional ROP seen in high-income countries with modern oxygen management, and ROP related to excessive oxygen supplementation in low- and middle-income countries: oxygen-associated ROP (OA-ROP).

## 1. Introduction

Retinopathy of prematurity (ROP) was first described in 1942 as an epidemic of blindness due to retrolental fibroplasia (RLF) among preterm infants, in which fibrous tissue formed behind the lens, the cause of which was originally unknown [1]. In what became known as the “first epidemic” of ROP [2], uncontrolled supplemental oxygen use was subsequently identified and confirmed as a major risk factor [3,4,5], and RLF nearly disappeared with strict oxygen regulation [6].

For the next two decades, RLF remained a rare event until a “second epidemic” of ROP occurred with improved survival of extremely preterm infants in the 1970s [2]. In these fragile infants at the threshold of viability, careful oxygen management was able to reduce, but not eliminate, the risk of blinding ROP [7]. With simultaneous advances in indirect ophthalmoscopy allowing for improved visualization of the developing retina, a standardized classification system was developed. The first International Classification of ROP (ICROP), published in 1984, formalized the term ROP to describe more subtle pathology in the developing retina that occurred prior to the permanent cicatricial changes of RLF [8].

Since the quality of neonatal care has improved and neonatal mortality rates have decreased, ROP is a leading cause of preventable childhood blindness worldwide [9,10]. In Latin America and former socialist countries in Eastern Europe, more than 300,000 children have become blind or visually impaired due to ROP [11]. Similar to the “first epidemic” of ROP, infants in this “third epidemic” of ROP have higher birth weights and gestational ages than traditionally seen since the “second epidemic,” and most are managed with uncontrolled supplemental oxygen in the first few weeks of life [12]. In some areas, ROP in this “third epidemic” has also proven amenable to prevention with oxygen management, education, and policy changes [13,14,15].

It is now estimated that at least 15 million preterm infants are born per year, and 80% of these are born in low- and lower-middle-income countries (LMICs) such as India and sub-Saharan Africa (SSA) [16]. With the second-highest preterm birth rate in the world [16], SSA now represents a “new frontier” for ROP [12]. In 2010 it was estimated that more than 32,000 preterm infants who survived longer than one month were at risk for ROP in SSA [11], and that number is now likely much larger. To halt the expansion of this third epidemic of ROP across SSA, the Children’s Eye Foundation of the American Association for Pediatric Ophthalmology and Strabismus and the International Pediatric Ophthalmology and Strabismus Council initiated a program called: Stop Infant Blindness in Africa (SIBA).

In a recent survey, SIBA found that the overwhelming majority of neonatal units in SSA cannot provide blended oxygen [17,18]. With limited resources for oxygen management combined with increased survival of preterm infants, blindness from ROP has now been documented across 23 countries in SSA [19]. To address this new wave of ROP in SSA, SIBA has donated oxygen management supplies and ophthalmic equipment and sponsored sub-specialty training teams to establish African-led Centers of Focus for ROP teaching, where other doctors and nurses in SSA will come to train.

In the “first epidemic” of ROP in high-income countries (HIC) with uncontrolled oxygen supplementation, RLF was largely eradicated prior to the development of indirect ophthalmoscopy or the first International Classification of ROP [8]. Given similarities between the first and third epidemics of ROP [20], modern ophthalmic equipment allows us to better describe clinical findings of ROP related to unmonitored supplemental oxygen that remained undetected in the first epidemic. Previously, the impact of oxygen management on ROP has been explored in detail [6]. This perspective aims to discuss clinical differences in ROP that are associated with variations in oxygen management, including animal models of oxygen-induced retinopathy (OIR), traditional ROP findings seen in HIC, and characteristic retinal findings associated with excess oxygen in LMIC, for which we propose the term oxygen-associated ROP (OA-ROP).

## 2. Animal Models of ROP

### Oxygen-Induced Retinopathy

Understanding variations in animal models of ROP illuminates aspects of ROP pathophysiology that manifest differently worldwide. As animal models typically involve newborns rather than preterm animals, most animal models require simulation of a condition called oxygen-induced retinopathy (OIR) in term animals, many of whom have not completed retinal vascularization at birth but lack the typical comorbidities of human preterm infants [21].

The current biphasic pathophysiology model of ROP was first postulated after studying kittens whose retinas are not fully vascularized until three weeks after birth. Exposing kittens to high oxygen, an initial hyperoxic phase of retinal vascular constriction and “obliteration” of growing retinal vessels was observed, which appeared directly related to the degree of immature vascularization and the magnitude of oxygen exposure. This “vaso-obliteration” from direct oxygen damage in “Phase 1” set the stage for “Phase 2,” when the avascular retina became relatively hypoxic due to poor retinal perfusion and increased oxygen demand in maturing avascular retinal tissue. The response to retinal hypoxia was abnormal re-vascularization, neovascularization into the vitreous, and subsequent retinal detachment [22].

Although no animal model of OIR perfectly simulates human ROP, some species may be more closely aligned to certain features of ROP seen in humans. For example, in the mouse model, while retinal vascularization is already complete when pups are placed into a continuous high-oxygen environment, direct oxygen damage from hyperoxia causes vaso-obliteration, followed by intravitreal neovascularization, similar to what was seen in humans in the first and third epidemics of ROP [21]. Obliteration of capillary beds is likely the cause of OA-ROP related to excessive oxygen supplementation currently seen in LMIC.

In contrast, in the rat model, intermittent hyperoxia leads to delayed retinal vascularization, and vaso-attenuation is seen rather than vaso-obliteration of existing vessels. This model of vaso-attenuation may more closely align with the typical ROP currently seen in HIC with improved oxygen management. Unlike ROP seen in humans, however, neither model typically progresses to retinal detachment [21].

As described by McLeod et al., the canine model shares many features of human ROP [23]. In this model, vaso-obliteration initially involves the most immature vessels, but continued exposure to hyperoxia leads to the progressive involvement of more mature vascular beds. After four days of hyperoxia, extreme vaso-constriction and vaso-obliteration resulted in capillary islands. When the capillary beds were obliterated but veins remained intact, arteriovenous shunts developed [23]. These arteriovenous shunts and capillary islands are also found in OA-ROP related to excessive oxygen supplementation currently seen in LMICs [20].

Although vasoconstriction has been more commonly used to describe typical ROP findings seen in HIC since the “second epidemic“ [6], the concept of vaso-obliteration may be useful in OA-ROP related to excessive oxygen supplementation currently seen in LMIC.

## 3. Clinical Manifestations of ROP

### 3.1. Traditional ROP in Extremely Preterm Infants with Strict Oxygen Regulation

Similar to the animal models of OIR, the pathophysiology of ROP in preterm human infants also appears biphasic. Even without supplemental oxygen, the retinal environment of the preterm human infant at birth is relatively hyperoxic compared to the intrauterine environment [24]. Supplemental oxygen accentuates the hyperoxic effect, suppressing growth factors such as vascular endothelial growth factor (VEGF) and erythropoietin. Loss of the maternal source of insulin-like growth factor 1 (IGF-1) during the third-trimester compounds this effect [7], as IGF-1 enables normal VEGF signaling for blood vessel growth [25]. Eventually, as retinal vessel growth stalls in Phase 1, the metabolic demands of the poorly vascularized retina eventually exceed the supply of oxygen available. As a consequence of poor vascularization in Phase 1, local hypoxia-driven increases in VEGF lead to pathologic neovascularization in Phase 2 [7]. Compounding this effect, IGF-1 levels rise with infant maturation [25].

Despite decades of clinical trials regarding neonatal oxygen support, no consensus on the optimal level of oxygen supplementation/saturation has been achieved to date to decrease the incidence of ROP while minimizing other adverse events [26]. After the first epidemic of ROP in developed countries, attempts to restrict oxygen dramatically decreased the occurrence of RLF but also increased mortality [6]; per case of blindness averted, inadequate oxygen supplementation resulted in about sixteen deaths [27]. More recently, a 2018 meta-analysis of five randomized control trials, including data from almost 5000 infants, found that lower oxygen saturation was associated with a lower risk of needing ROP treatment but a higher risk of necrotizing enterocolitis and death [28], with one additional death for every two cases of severe ROP averted [29]. Particularly, in an era of effective ROP treatment options, oxygen goals should be patient-specific, taking into consideration multiple neonatal comorbidities that may differentially affect an individual preterm infant.

Even with ideal oxygen management, some extremely preterm infants will still develop severe ROP [7]. In HIC with strict oxygen management protocols, most infants with a birthweight <1250 g develop some ROP, but in the majority ROP regresses spontaneously [30,31]. Despite strict oxygen management, however, extremely preterm infants remain at high risk of developing severe ROP, particularly those with birthweights below 750 g and gestational ages below 27 weeks [31].

For infants who developed severe ROP, treatment was first shown to prevent retinal detachment in the 1988 cryotherapy for ROP study [32], with such impressive results that the trial was terminated early as it was felt unethical to withhold such an effective therapy. From 1988 to 2002, “Threshold disease” defined the severity of ROP at which treatment reduced the chance of retinal detachment and adverse retinal structural outcomes by 50%. Gradually, photocoagulation, laser treatment to the peripheral avascular retina, replaced cryotherapy since it was shown to be less stressful and at least as effective as cryotherapy [33,34]. In 2002, the Early Treatment for ROP (ET-ROP) study showed that earlier treatment of patients with “Type 1 ROP” resulted in better structural and visual outcomes compared to Conventional Treatment at “Threshold disease” [35]. Type 1 ROP (zone I, stage 3 with or without plus; zone I, any stage with plus; zone II, stage 2 or 3 with plus) remains the current treatment criteria for ROP today.

Since 2011, treatment of ROP using intravitreal injections of anti-VEGF agents have further improved structural outcomes for infants with posterior stage 3 ROP in the presence of plus disease [36] or aggressive posterior ROP (AP-ROP) [37]. Although visual and refractive outcomes are also likely improved with anti-VEGF compared to laser treatment [38,39,40], ROP reactivation leading to retinal detachment and adverse retinal structural outcomes may occur well outside the timeframe of standard screening guidelines [41]. Given numerous reports of ROP reactivation occurring even ten years after the initial anti-VEGF treatment [42,43,44,45,46], with the potential for progressive, atypical retinal detachments [47], many recommend prophylactic laser treatment to peripheral avascular retina following anti-VEGF treatment [48]. When laser treatment is applied after 60 weeks’ PMA, it does not appear to undermine the refractive benefits of primary bevacizumab treatment [40].

**Figure 1:**
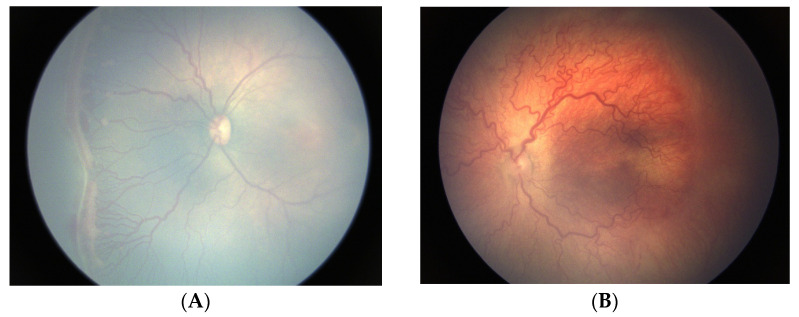
Patients with typical retinopathy of prematurity (ROP) requiring treatment (Type 1 ROP) in a neonatal intensive care unit with modern strict oxygen regulation. (**A**) Preterm infant with gestational age of 26 weeks and birthweight of 600 g, who developed stage 3 ROP in zone I (Type 1 ROP) at 38 weeks’ post-menstrual age. Notice the traditional stage 3 ROP, characterized by extraretinal neovascular proliferation. (**B**) Fundus photograph of a baby born at 24 weeks and a birthweight of 710 g with very severe bronchopulmonary dysplasia. As pictured at 34 weeks’ post-menstrual age, note the deceptively featureless networks of flat neovascularization in the setting of severe plus disease and aggressive ROP (A-ROP).

Figure 1 illustrates typical treatment-warranted (Type 1) ROP in extremely preterm infants despite judicious oxygen regulation in one HIC. These typical ROP patients were born at 24–26 weeks’ gestational age, with birthweights of <750 g, and suffered multiple comorbidities, including severe bronchopulmonary dysplasia. The first infant has stage 3 in zone I (Figure 1A), classic for Type 1 ROP as defined by the ET-ROP study [35]. The second infant has “dilation and tortuosity in all four quadrants that are out of proportion to the peripheral retinopathy,” exemplifying what was formerly called AP-ROP by the second International Classification of ROP (ICROP-revisited) [49].

### 3.2. Oxygen-Associated ROP Seen in Larger Preterm Infants

In contrast to the extremely small preterm infants who develop severe ROP in HIC despite judicious oxygen use, relatively large infants can lose previously vascularized retinas when exposed to 100% oxygen [20]. Although such large infants would be assumed to have a low risk of developing ROP in HICs, babies over 34 weeks’ gestation with birthweights of 2500 g have been blinded from ROP with unregulated oxygen supplementation in LMIC [50,51].

While the demographics of infants with ROP vary worldwide depending on prenatal care and oxygen management [21], distinct clinical findings on the retinal examination have been described. In recognition of rapidly progressive ROP that extends beyond the posterior retina, most commonly among larger/older infants exposed to excess oxygen [20], the Third Edition of ICROP (ICROP3) [52] replaced the term AP-ROP with A-ROP to take the focus away from the posterior location of disease to the tempo of development of this rapidly progressive form of ROP. This type of ROP is classically described in parts of the world with limited resources, where infants are often exposed to excess unmonitored supplemental oxygen [20].

In addition to A-ROP, obliteration of vessels posteriorly, more anterior shunt vessels, capillary non-perfusion, and islands of avascular retina within vascularized retina have also been described [20]. Following excess unmonitored supplemental oxygen, infants can lose previously vascularized retinas and develop shunt vessels. In a cohort of 99 larger preterm infants exposed to 100% oxygen in India, 75% of patients had loss of vascularization. This cohort had a mean gestational age of 31.7 weeks and a mean birthweight of 1572 g, criteria by which such infants would not qualify for ROP screening in many HIC. Compared to the initial examination in which most infants had vascularization into zone II, with a few vascularized into zone III, all patients developed severe plus disease with avascular retina extending into zone I at subsequent examinations four weeks later. In addition, large looping shunt vessels were also noted extending anteriorly. The avascular islands and shunt vessels are similar to those seen in the canine model [23]. The authors highlighted epidemiologic similarities with the “first epidemic” of ROP [20].

Similarly, “ROP-like” findings have also been described in allegedly low-risk infants (mean gestational age 33.7 weeks and mean birth weight 1848 g) exposed to 100% oxygen in Mexico. In 28 eyes of 15 infants who underwent fluorescein angiography, the authors described two groups demonstrating “ROP-like” retinal pathology. The first included nonproliferative retinopathy with capillary non-perfusion, arteriovenous shunting, plus-like disease, and absence of the foveal avascular zone (FAZ) without leakage of dye. The second was proliferative retinopathy with leakage of dye at the border between the vascular and avascular retina and/or at the optic disc. In the first group, the authors hypothesized that lack of dye leakage, which is common in typical ROP, suggests direct damage to more mature vessels rather than a primary defect of angiogenesis [53]. Similarly, capillary dropout may be more likely the result of the obliteration of previously formed vessels, similar to the OIR described in animal models [53].

**Figure 2:**
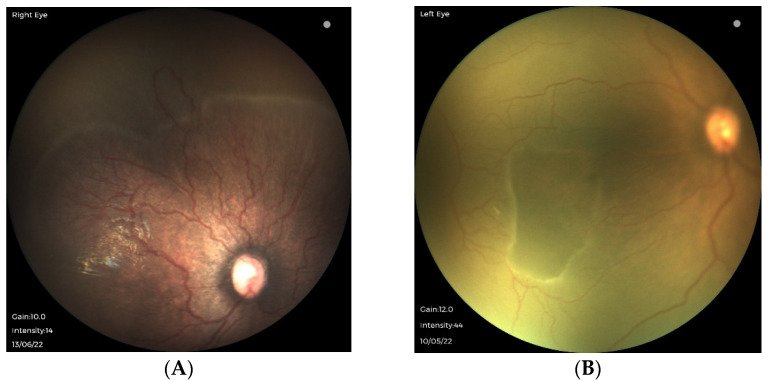
Retinal pathology photographed at 36 weeks’ post-menstrual age in a preterm infant born at 32 weeks and 1500 g, exposed to 100% oxygen. (**A**) The right eye has shunt vessels extending beyond the vascular–avascular junction superiorly. (**B**) The left eye has an island of avascular retina within otherwise vascularized retina nasally.

**Figure 3:**
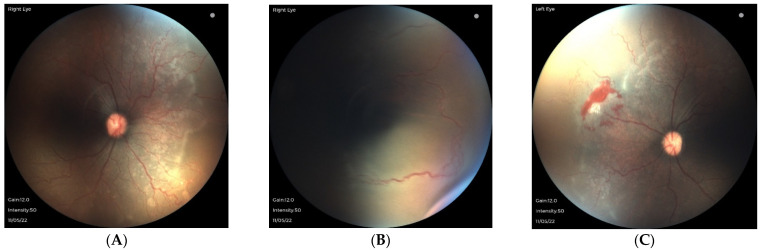
Fundus photograph at 36 3/7 weeks’ post-menstrual age in a larger infant (gestational age 32 weeks, birthweight 1600 g), exposed to 100% oxygen. (**A**,**B**) Notice the posterior vessel changes with areas of fibrosis and large looping vessels with capillary dropout. Although the avascular retina extends into zone I, large looping vessels can be seen extending more anteriorly into the nasal periphery surrounding areas of capillary dropout. (**C**) Similar findings are seen in the left eye with fibrosis and shunt vessels extending peripherally. Note the flat neovascularization and fibrosis, which are distinct from the traditional stage 3 retinopathy of prematurity of extraretinal neovascularization shown in Figure 1A.

Following excess unmonitored supplemental oxygen, unusual features of ROP have also been observed in larger preterm infants in SSA. After exposure to 100% oxygen, infants born at 32 weeks’ gestational age with birthweights ≥1500 g have developed distinctive retinal pathology not specifically described in ICROP. As shown in Figure 2 and Figure 3, vascular pathology is observed posterior to the vascular/avascular junction, including islands of the avascular retina within the vascularized retina. Large looping shunt vessels are noted more anteriorly, even into areas of the avascular retina, along with areas of capillary non-perfusion and areas of fibrosis. Because these characteristic retinal findings are associated with excess unmonitored supplemental oxygen, we propose the term oxygen-associated ROP, or OA-ROP.

Across LMICs, including India, Mexico, and SSA, these characteristic features of ROP seen, including capillary non-perfusion, dilated vascular loops, and arteriovenous shunts, seem to indicate vascular damage rather than a primary defect of vasculogenesis [54]. In particular, the loss of previously vascularized retina (whether loss of larger vessels into zone I or loss of capillaries leading to non-perfusion or avascular islands) seems to reflect oxygen damage following excess unmonitored supplemental oxygen.

While primary prevention of preterm birth is the most important factor in reducing the prevalence of severe ROP, careful oxygen regulation is critical in minimizing the risk of blindness due to ROP among very preterm and extremely preterm infants [13]. To this end, SIBA is sending oxygen blenders and sensors to neonatal units throughout SSA and training nurses to use them properly at African-led Centers of Focus. However, even with meticulous oxygen management, the risk of ROP cannot be eliminated in extremely preterm infants [7], and some infants will still require treatment. Meanwhile, recognizing characteristic findings of OA-ROP may improve identification of intants who would benefit from treatment.

## 4. Future Directions

Most major clinical trials for ROP treatment [32,35,36] have been conducted in the setting of traditional ROP in HIC. Just as screening guidelines should be country specific [2], treatment criteria and techniques validated for traditional ROP findings may need to be adapted for the different characteristic ROP findings noted in LMIC. For example, islands of avascular retina seen following excess unmonitored supplemental oxygen in LMICs may improve without treatment. Even in larger babies who develop ROP related to high and uncontrolled supplemental oxygen exposure, abnormal findings have been shown to reverse spontaneously [20]. Given that studies such as ET-ROP did not include these atypical cases, more studies are needed to determine the optimal timing and modality of treatment of OA-ROP findings in LMIC.

In contrast to HIC, where many patients remain in the neonatal unit at the time of ROP treatment, patients in LMICs are frequently discharged home prior to developing severe ROP, and practical considerations must be taken into consideration when determining the timing and modality of treatment. Laser, a more definitive treatment than anti-VEGF treatment, may be most appropriate in circumstances where follow-up is uncertain, although readmission to the neonatal unit for sedation and apnea monitoring poses financial and logistical challenges. On the other hand, OA-ROP patients with diffuse posterior capillary loss and severe ischemia may fare better with anti-VEGF than laser treatment [20]. When parents may be responsible for the entire cost of examinations and treatment, however, anti-VEGF agents must be made affordable. Although vessels may continue to grow anteriorly with improved capillary perfusion after anti-VEGF treatment [20], reactivation rates after anti-VEGF are unknown in this setting. Even in HIC, where anti-VEGF agents have been used to treat ROP for over a decade, controversy regarding the optimal treatment remains. More long-term data comparing anti-VEGF to laser treatment for ROP are needed worldwide in various locales and in the setting of various ROP findings.

Finally, recent studies in HIC have shown the potential to reduce the progression of ROP with biphasic oxygen targeting, although evidence for these protocols is lacking in LMIC. During the first epidemic of ROP, Thaddeus Szewczyk observed that rapid withdrawal of oxygen was associated with retinal venous engorgement [55]. In a subset of patients without plus disease in the Supplemental Therapeutic Oxygen for Prethreshold ROP (STOP-ROP) study, patients with pre-threshold disease but no plus were less likely to progress to threshold disease when given supplemental oxygen [56]. Subsequent before-and-after retrospective studies have suggested that lower oxygen targets in Phase 1 with higher oxygen targets in Phase 2 may decrease the rate of severe ROP [57,58]. Although the preventative role of biphasic oxygen targeting needs validation in LMIC, slow and careful weaning of infants still on oxygen during Phase 2 may be beneficial, particularly if ischemia is present. However, many of these infants in LMIC may be treated and examined in the outpatient setting, and readmission to the neonatal unit for oxygen management may not be feasible. As with treatment options for severe ROP, specific oxygen targeting after hospital discharge, in hopes of preventing progression to severe ROP, may also be limited by practical considerations.

## 5. Conclusions

In conclusion, over 80 years and three epidemics, with the third epidemic now expanding across SSA, the complex interaction between oxygen and the developing retina continues to present new challenges. When RLF was first described, the indirect ophthalmoscope was not yet available, and RLF was eradicated prior to the first International Classification of ROP. Modern ophthalmic equipment (indirect ophthalmoscopy, retinal cameras, fluorescein angiography, and ocular coherence tomography) now offers an unprecedented window into the retina, which was not previously available during the first epidemic of ROP, and allows us to better describe characteristic features of OA-ROP, which may have been present but undocumented in those early cases of RLF. As SIBA is working to halt another wave of blinding ROP across SSA, we should consider adapting our current strategies for ROP management and validating them in the setting of OA-ROP to facilitate the identification of infants with potentially blinding disease. In the future, a consensus report specific to OA-ROP may be helpful.
